# Timed microglia depletion promotes functional network reorganization and motor recovery after stroke

**DOI:** 10.1093/braincomms/fcag202

**Published:** 2026-06-02

**Authors:** Sara Isla Cainzos, Fanny Quandt, Malte Borggrewe, Hanna-Marie Altjohann, Tim Magnus, Jonatan Biskamp

**Affiliations:** Department of Neurology, University Medical Center Hamburg-Eppendorf, Hamburg 20246, Germany; Department of Neurology, University Medical Center Hamburg-Eppendorf, Hamburg 20246, Germany; Independent Bioinformatics Consultant, Hamburg 20259, Germany; Department of Neurology, University Medical Center Hamburg-Eppendorf, Hamburg 20246, Germany; Department of Neurology, University Medical Center Hamburg-Eppendorf, Hamburg 20246, Germany; Department of Neurology, University Medical Center Hamburg-Eppendorf, Hamburg 20246, Germany

**Keywords:** ischaemic stroke, CSF-1R inhibitor, microglial repopulation, sensorimotor networks, motor rehabilitation

## Abstract

Pharmacological options to promote long-term rehabilitation after stroke remain limited. We investigated how short-term microglia depletion during the subacute phase after ischaemic stroke affects functional recovery and neuronal network dynamics. Using a C57BL/6 wild-type stroke model, fine motor performance was assessed with a skilled reaching task while bilateral cortical activity was recorded longitudinally through epidural electrocorticography. Microglia were depleted between days 3 and 7 post-stroke using PLX5622, a colony-stimulating factor 1 receptor inhibitor. Microglia depletion resulted in near-complete restoration of fine motor function by day 7 and was accompanied by significant functional connectivity changes in bilateral sensorimotor networks, including increased beta-band connectivity in the ipsilesional motor cortex, which correlated with contralateral fine motor improvement. Following repopulation, microglial cells showed altered morphology and gene expression profiles. These findings reveal that transient microglial modulation during the subacute phase after stroke promotes cortical network reorganization and motor recovery.

## Teaser

Sara *et al*. show that transient microglial depletion during the subacute phase after cortical stroke promotes motor recovery and sensorimotor network reorganization. These findings highlight the role of microglia in shaping post-stroke recovery trajectories and suggest that temporally controlled modulation of microglial activity may support functional recovery.

## Graphical Abstract

**Graphical Abstract:**
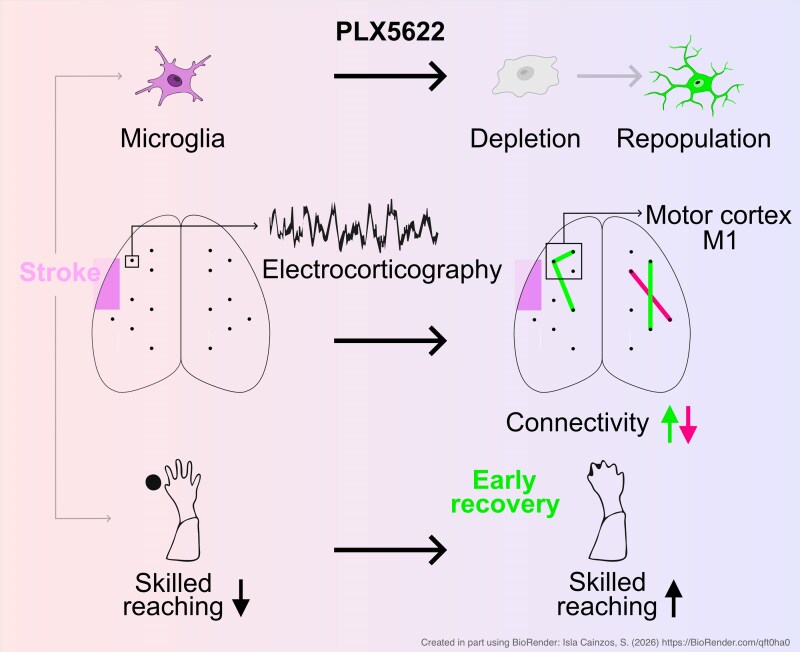

## Introduction

The global burden of stroke continues to rise, with nearly 12 million new cases and over 7 million deaths annually.^1^ It results from sudden disruption of cerebral blood flow, triggering neuroinflammation, oxidative stress and dysfunction of the blood-brain barrier, which drive both immediate and delayed brain damage.^2,3^ Although acute recanalization therapies have transformed stroke care, their use remains limited by narrow time windows and strict eligibility criteria, leaving many patients without targeted acute intervention.^4,5^ Pharmacological options that influence the subsequent disease course and support long-term recovery remain limited, underscoring the need for therapies that promote recovery beyond the acute phase and for a deeper understanding of the mechanisms underlying stroke repair.^6,7^

To address this gap, the brain’s innate immune system has emerged as a potential modulator of recovery. In particular, microglia, the resident immune cells of the central nervous system, play a pivotal role, initially promoting inflammation, later contributing to tissue repair and recovery.^8^ Microglia engage in diverse interactions with neurons, continuously surveying their environment through dynamic cellular processes and participating in the remodelling of synaptic connections.^9^ Moreover, their ability to shape synaptic receptor composition and modulate network oscillations both in vitro and in vivo has been demonstrated.^2,10-13^

Over the past decade, strategies aimed at depleting microglia have emerged as promising translational approaches for various neurological disorders.^14^ Microglia survival critically depends on signalling through the colony-stimulating factor 1 receptor (CSF-1R).^15^ The small molecule PLX5622 selectively antagonizes CSF-1R, resulting in efficient microglial depletion and, upon withdrawal, repopulation of the microglial compartment.^15,16^

Recent studies have demonstrated encouraging outcomes following short-term CSF-1R inhibition in models of neuronal injury. For instance, microglial renewal in a murine model reduced lesion-induced release of inflammatory markers, resolved the activated microglial phenotype, and improved behavioural deficits.^6^ Similarly, transient depletion followed by repopulation of microglia after traumatic brain injury or ischaemia mitigated neuropathological changes and neurological impairments, with short-term CSF-1R inhibition promoting microglial repopulation and associated changes in glial morphology, phenotype, and gene expression, correlating with enhanced functional recovery.^17-19^

Based on these findings, we hypothesize that short-term microglial depletion in the subacute phase after ischaemic stroke, followed by repopulation, significantly impacts neuronal activity within the penumbra and promotes functional recovery. To assess this, we evaluated changes in neuronal network activity across the acute injury phase and the subsequent recovery period using a 16-channel electrocorticography (ECoG) electrode array implanted over both hemispheres. Functional recovery was measured using the single-pellet reaching task (SPR), allowing us to track fine motor deficits over time. Given that the primary motor cortex (M1) plays a critical role in mediating recovery following sensory cortical strokes,^20^ we placed particular emphasis on M1 activity. PLX5622 was administered daily from day 3 to day 7.

Despite no significant differences in infarct size between groups, mice treated with PLX5622 showed near-complete recovery of fine motor function as early as day 7, coinciding with the peak of microglial depletion. Recovery of contralateral forepaw function was paralleled by pronounced changes in bilateral sensorimotor networks, most notably a sustained increase in beta-band connectivity within the ipsilesional motor cortex and a decrease in contralesional somatosensory gamma connectivity. Following repopulation, microglia displayed altered morphology and a distinct transcriptomic profile, while behavioural improvements remained stable. With this study, we identified transient microglial depletion as a therapeutic strategy capable of cortical network reorganization and supporting post-stroke motor recovery.

## Materials and methods

### Ethics statement

C57Bl/6 male mice aged nine postnatal weeks at the start of the experiment provided by the animal facility of the University Medical Center Hamburg-Eppendorf were used in this study. Experimental protocols were approved by the Behörde für Justiz und Verbraucherschutz der Freien und Hansestadt Hamburg (approval numbers N53/2020 and N102/2023). All procedures followed the guidelines of the animal facility of the University Medical Center Hamburg-Eppendorf and complied with the *Guide for the Care and Use of Laboratory Animals*. Animals were housed in groups under a 12 h dark-light cycle. After implantation of electrodes, mice were housed individually for 49 days.

Details on study design and the following methods are in the Supplementary material.

### Study design

**Figure 1:**
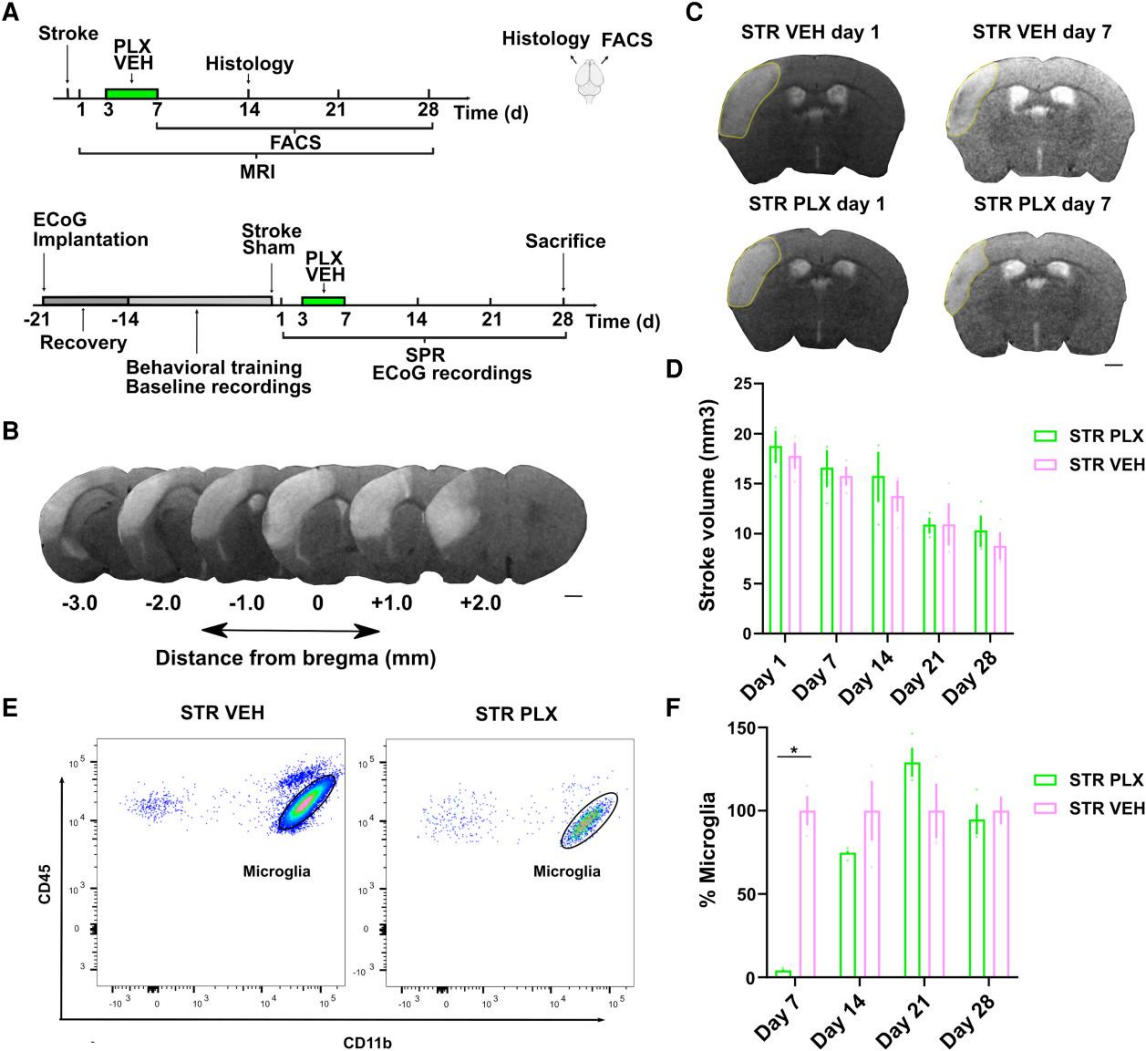
**Short-term PLX5622 treatment induces microglia depletion after stroke, followed by repopulation upon drug withdrawal.** (**A**) Timelines of the experiment. (**B**) Representative coronal brain MRI sections after stroke. (**C**) Representative illustration of stroke volume differences between PLX and VEH groups; lesion area indicated by an outline. (**D**) Stroke volume quantification. STR PLX: *n* = 3; STR VEH: *n* = 3. Two-way ANOVA with Sidak’s multiple comparisons test. (**E**) Gating strategy used to assess microglial depletion. (**F**) Percentage of microglia remaining after treatment. STR PLX: *n* = 3; STR VEH: *n* = 3. Mixed-effects model (REML) with Sidak’s multiple comparisons test. Black scale bars in **B** and **C** indicate 1 mm. *P < 0.05. Data are shown ± SEM. Each data point represents a single mouse. PLX, PLX5622; SH, sham; STR, stroke; VEH, vehicle.

To assess microglial depletion, repopulation and morphology as well as stroke volume a cohort of 24 wild-type mice underwent dMCAo surgery and received either daily PLX5622 or vehicle treatment from day 3 to day 7 post-stroke. Flow cytometry (FACS) was performed on the contralesional hemisphere at days 7, 14, 21, and 28 post-ischaemia (pi). Histological analysis on the ipsilesional hemisphere was performed at day 14 pi. Stroke volume was assessed at days 1, 7, 14, 21 and 28 pi with MRI (Fig. 1A).

To investigate the effects of microglia depletion and repopulation on functional deficits and recovery after stroke a cohort of 49 wild-type mice were implanted with electrocorticography (ECoG) electrodes and trained in a single-pellet reaching task (SPR). After dMCAo or sham surgery mice received daily PLX5622 or vehicle treatment from day 3 to day 7 pi. Mice were functionally tested and recorded at days 1, 3, 7, 14, 21, and 28 pi. Before any behavioural training or electrophysiological recording started mice were granted a recovery period of one week after electrode implantation (Fig. 1A).

To study the effects of microglia depletion and repopulation at a phenotype level, a third cohort of 8 wild-type mice underwent dMCAo surgery and received either PLX5622 or vehicle treatment from days 3 to 7 pi. Single-cell RNA sequencing analysis was performed at day 14 pi on pooled samples within each treatment group.

### Electrode implantation and dMCAo surgery

Electrode implantation and dMCAo surgery were performed as previously described.^20^ In short, 16 platinum-iridium electrodes of 127 μm diameter were chronically implanted covering both hemispheres (see Supplementary Table 1 for stereotactic coordinates), and then a permanent dMCAo without reperfusion was induced contralateral to the dominant forepaw or randomly on either hemisphere if no paw preference could be determined. As previously shown, ischaemic lesions were located exclusively within the sensory cortex (Fig. 1B and C).^20^

### PLX5622 or vehicle treatment

PLX5622 (MedChemExpress, HY-114153) was administered for four consecutive days, starting on day 3 after dMCAo or sham surgery. The compound was prepared at a concentration of 1200 ppm by dissolving PLX5622 in a 10% DMSO/90% corn oil solution and then mixed with soft food. Mice received the PLX5622-containing soft food in a Petri dish twice daily at 10–12 h intervals for voluntary oral intake. Body weight was monitored daily, and the provided food was consistently fully or near-fully consumed. A vehicle control group received soft food prepared with 10% DMSO/90% corn oil without PLX5622.

### Infarct volume analysis using MRI

Infarct volume was assessed using a 7-Tesla small animal MR imaging system (ClinScan, Bruker, Ettlingen, Germany) with a T2-weighted MRI protocol. Infarct volumes were quantified using NIH ImageJ software (version 1.53e). Corrected stroke volumes, accounting for edema-related tissue swelling to avoid overestimation of infarct size, were calculated as previously described.^21^

### Tissue isolation, FACS, histology and microglia sorting

At given time points animals were terminally anesthetized with ketamine/xylazine (120 and 16 mg/kg, respectively) and intracardially perfused with 20 mL of 0.1 M PBS. Brains were removed and dissected.

For FACS, the contralateral hemisphere was enzymatically digested in Dulbecco’s Modified Eagle’s Medium (DMEM) containing Collagenase (1.0 mg/mL, Sigma, 11088793001) and DNase (0.1 mg/mL, Sigma, 11284932001) for 30 min at 37°C. The digested tissue was filtered through a 40 µm strainer, followed by erythrocyte lysis and Percoll gradient centrifugation (Cytiva, 17-5445-01) to isolate immune cells. For extracellular staining, cells were incubated with a pre-prepared antibody cocktail [CD45-V421 (BioLegend, 103134), CD11b-PECY7 (BioLegend, 101216), and P2Y12-APC (BioLegend, 848006), along with Fc-block and normal rabbit serum (NRS)] all at 1:100 dilutions for 30 min at 4°C. After washing, stained cells were resuspended in 250 µL FACS buffer and analyzed using flow cytometry within four hours. BD Trucount tubes (BD Biosciences, 663028) were used for quantitative analysis. Data were acquired using BD FACSCANTO II (BD Biosciences, Franklin Lakes, NJ) and analyzed with FlowJo software (Tree Star, Ashland, OR) (see Supplementary Fig. 1).

For histology, the ipsilesional hemisphere was post-fixed in 4% PFA, rinsed in PBS for 24 h at 4°C, dehydrated in a graded ethanol series, embedded into paraffin blocks and coronally sectioned (5 μm slice thickness, 500 μm intervals) using a microtome. Immunohistochemistry was performed using anti-Iba1 (Rabbit, Wako Chemicals, 013-27691) to label microglia, and sections were imaged using a Zeiss MP In Vivo Platform. 10 to 14 microglial cells in the penumbra area were analyzed per animal. Fractal and Sholl analyses were performed in Fiji/ImageJ2. Images were pre-processed automatically, with manual tracing of soma and ramifications.^22^ Fractal-Analysis was performed using the *FracLac* plugin (Version 2.5) for ImageJ.^23^ Sholl-Analysis was performed with the *Sholl-Analysis* plugin (Version 4.3.0) included in the *SNT Neuroanatomy* battery for ImageJ.^24^

For single-cell sequencing, mice were terminally anesthetized as described above, with PBS containing Actinomycin D (5 µg/mL, Carl Roth, 8969.1) and Triptolide (10 µM, Thermo Fisher, 466840250) added during perfusion to inhibit gene expression during tissue processing. Brains were removed, and the ipsilesional cortex dissected and digested in Papain solution (Worthington, LK003176) containing Actinomycin D (5 µg/mL), Triptolide (10 µM), and Anisomycin (27.1 µg/mL, Sigma-Aldrich, A9789) for 20 min at 37°C. This was followed by further digestion with Collagenase/DNase solution (containing the same inhibitors) for 10 min. The digested tissue was filtered through a 100 µm strainer and subjected to density gradient centrifugation using Percoll (Cytiva, 17-5445-01). The resulting pellet was resuspended in FACS buffer (PBS with 1% fetal bovine serum) and stained with antibodies against CD11b-PE-Cy7 (BioLegend, 101205), CD45-BV510 (BioLegend, 103137), and near-infrared APC-Cy7 (Thermo Fisher) to label microglia, along with Fc block and near-infrared viability dye (all at 1:100 dilutions). Microglia was then sorted using a FACS AriaFusion sorter (BD Biosciences, Franklin Lakes, NJ).

### Single-cell sequencing analysis

Raw reads were processed with the nf-core/scRNAseq pipeline (v4.0.0) using Cell Ranger (v8.0.0) for alignment against the 10× Genomics mouse reference (GRCm39, release 2024-A).^25^ Data were analyzed using Scanpy (v1.11.1).^26^ Low quality cells and potential doublets were filtered based on quality control thresholds (mitochondrial reads <5%; number of genes between 750 and 5000), followed by count normalization (sc.pp.normalize_total, sc.pp.log1p) and highly variable genes selection (sc.pp.highly_variable_genes based on seurat_v3 method and top 2000 variable genes). The uniform manifold approximation and projection (UMAP) based on the first 10 principal components was computed and after initial Leiden clustering, two small clusters (∼200 cells total) formed based on low number of genes, which were removed. Leiden clustering was used to define cell subsets, which were annotated based on known marker genes of microglia homeostatic and reactive microglia, as well as markers for other myeloid cells (see Supplementary Tables 2, 3 and 4). Differentially expressed genes in PLX5622 group compared with vehicle and in distinct homeostatic subsets were determined using the MAST framework (v1.32.0).^27^ Gene set activity was scored using AUCell via decoupleR (v2.0.5) and differential activity was determined using a Wilcoxon test. Gene set activity analyses were performed using previously published DAM-like and repair-associated gene sets as well as PLX-repopulation marker gene sets.^7,28^

### Behaviour

Fine motor skills were evaluated using a SPR task.^20^ In short, mice were placed in a transparent chamber and trained to reach through a window to retrieve a food pellet placed outside. During the first week of training, the dominant forepaw was determined. Then, individual baselines were defined for each mouse, averaging the number of successful reaching attempts on the last 2 days of training. Test performance was assessed in relation to individual baseline performance (in %). General locomotor activity was evaluated in an open field scenario using ANY-maze Video Tracking System 7.48 (Stoelting Co.).

### In vivo electrophysiology and data analysis

ECoG data were recorded from freely behaving mice (W2100 System, HS16 Headstage, Multichannel Systems).^20^ In short, mice were allowed to explore a circular arena while being videotaped (UI-3240-NIR-GL Rev.2, IDS Imaging; W2100-Video-System-NIR, Multichannel Systems). Episodes of locomotion (later termed ‘active’) as well as episodes of resting behaviour with a minimum duration of 2 s were visually identified offline and used for electrophysiological data analysis.

Data processing was performed in MATLAB using the FieldTrip toolbox.^29^ Continuous data were filtered between 0.3 and 180 Hz using a 4th-order Butterworth filter. Then, episodes corresponding to active behaviour were extracted and artifactual episodes rejected manually. Power spectral density (PSD) was then computed for each clean episode and averaged over time of each experiment. To that end, data were demeaned first, then Fast-Fourier Transformation after multi-tapering with 7 tapers was applied resulting in a spectral smoothing of ±2 Hz given the 2 s length of episodes. Peak frequencies of the PSD as well as aperiodic parameters were extracted in Python using the FOOOF toolbox (v1.0.0).^20,30^ For further analysis, frequency bands were defined as theta (4–10 Hz), low beta (10–20 Hz), high beta (20–30 Hz), and gamma (30–60 Hz). Relative power was calculated by dividing the power of given frequency bands by the total spectral power within the 1–60 Hz range.

For connectivity analysis, signals were re-referenced to the common average after excluding artifactual channels. Connectivity was computed as the imaginary part of the cross-spectral coherence, and spectra were averaged over time of each experiment. Animals with fewer than four artifact-free episodes in the channels of interest on day 1 were excluded from the analysis. Peak frequencies in the coherence spectra were determined visually. If the highest value within a frequency band corresponded to a peak outside the band, the next-highest peak within the band was selected.

For group-level comparisons, analyses were performed after matching anatomically corresponding channels between hemispheres. By convention, channels from the ipsilesional hemisphere were assigned odd numbers and are plotted on the left.

### Statistical analysis

Statistics were performed in Prism (9.3.0, GraphPad) and R (4.1.1, RStudio). For four-group comparisons (body weight and open field), two-way repeated measures ANOVA followed by Tukey’s multiple comparisons test was used. When values were missing at random (SPR task), mixed-effects model (REML) with Tukey’s multiple comparisons was used. For two-group comparisons (stroke volume) two-way ANOVA with Sidak’s multiple comparisons was used, while datasets with missing values at random (percentage of microglia) were analyzed using a mixed-effects model (REML) with Sidak’s multiple comparisons. Ordinary one-way ANOVA with Tukey’s multiple comparisons was used to assess weight and age at the time of intervention and a contingency analysis was performed to evaluate the lateralization of dMCAo and sham procedures. Assumptions of normality and equal variance were assessed for all parametric tests. Specifically, for cell morphology and recovery ratio normality was assessed by the Shapiro-Wilk test; nested *t*-tests were used for normally distributed data and Mann–Whitney tests for non-normally distributed data. Data are presented as mean ± SEM and were considered statistically significant when *P* < 0.05.

## Results

To assess the effect of short-term microglia depletion and repopulation on functional recovery and neural activity after sensory cortical stroke, mice received either the compound PLX5622 (PLX) or vehicle (VEH) treatment between day 3 and day 7 after dMCAo or sham surgery (Fig. 1A).

MRI scans at day 1, 7, 14, 21, and 28 post-dMCAo revealed no significant differences in lesion volume between PLX and vehicle treated animals (Fig. 1B-D). At the group level, stroke volumes decreased from an initial ∼18 mm^3^ at day 1 to ∼10 mm^3^ by day 28. In conclusion, PLX administration between day 3 and 7 after stroke was not associated with detectable differences in gross lesion volume over time.

Flow cytometry analysis confirmed >95% depletion of microglia by day 7 after stroke in PLX-treated animals [mean difference to VEH = −152.22, 95% CI (−273.00 to −31.44), adjusted *P* = 0.032]. After treatment withdrawal, microglia numbers gradually repopulated (day 14, ∼75%, *P* = 0.754; day 21, ∼130%, *P* = 0.617; day 28; ∼100%, *P* = 0.991; Fig. 1E and F).

**Figure 2:**
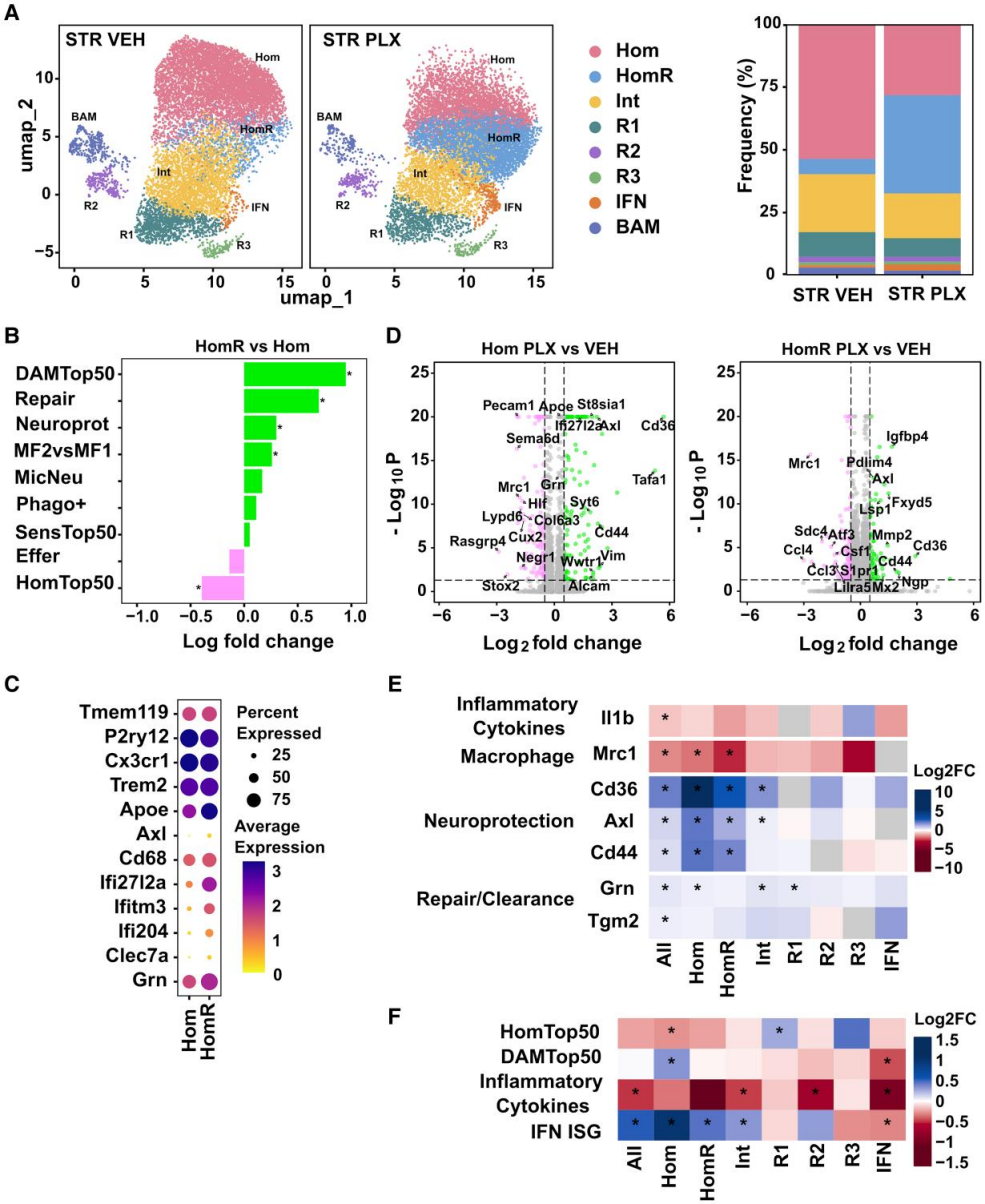
**Single-cell RNA sequencing of repopulated microglia at day 14 post-stroke.** (**A**) Left, separate UMAPs per condition showing microglia clustering. Right, frequency (%) of cells in each cluster. STR PLX: 10 378 cells; STR VEH: 10 744 cells (pooled from *n* = 4 mice per group). (**B**) Bar graph of differential GSA comparing HomR versus Hom; analysis performed at the single-cell level (same dataset as in (**A**). (**C**) Dotplot of selected genes upregulated in the PLX group. (**D**) Volcano plots of differentially expressed genes (DEGs) in selected clusters. (**E**) Heatmap of selected DEGs across clusters. (**F**) Heatmap of differential gene set activity (GSA) comparing PLX versus VEH; analysis performed at the single-cell level using a Wilcoxon test. STR, stroke; PLX, PLX5622; VEH, vehicle; Hom, homeostatic; HomR, homeostatic Reactive; Int, intermediate; R1–R3, reactive subtypes 1–3; IFN, IFN-responsive; BAM, border-associated macrophages; DAMtop50, disease-associated microglia top 50 genes; Neuroprot, neuroprotection; MF1/MF2, macrophage subtype 1 and 2; MicNeu, microglia-neuron interaction; Phago+, positive regulation of phagocytosis; SensTop50, sensome top 50 genes; Effer, efferocytosis; HomTop50, homeostatic top 50 genes; IFN_ISG, interferon-stimulated genes; *significantly differential gene expression (C–E: log2FC > 0.5, *P-*adjusted < 0.05) or GSA activity (B, F: log2FC > 0.25, *P-*adjusted <0.05).

Single-cell RNA sequencing of microglia from the ipsilateral cortex at day 14 after dMCAo revealed that repopulated microglia exhibited an altered transcriptional pattern compared with vehicle-treated samples (Fig. 2). Cells were clustered and annotated based on established marker genes (Supplementary Fig. 2A) and gene set activity related to microglial functions (Supplementary Fig. 2B). In the repopulated group, a population of homeostatic microglia with more reactive transcriptional features (HomR) was identified (Fig. 2A). This population maintained homeostatic marker expression but exhibited increased activity of gene sets associated with disease-associated microglia (DAM), neuroprotection, and tissue repair (Fig. 2B), and enhanced expression of reactive genes (*Apoe*, *Axl*, IFN-response) and chemokines (Fig. 2C, Supplementary Fig. 2C). Comparing PLX with VEH, in the homeostatic compartment both the classical homeostatic cluster (Hom) and HomR showed elevated expression of genes involved in phagocytosis, tissue remodelling, and neuroprotection (*Cd36, Axl, Cd44*), with HomR showing a tendency toward increased expression of repair and debris clearance genes (*Grn, Tgm2*) (Fig. 2D and E). Pro-inflammatory cytokines, such as *Il1b*, and cytokine-related gene sets were decreased across microglia in the PLX group, albeit not significant for all subclusters (Fig. 2E and F). In contrast, most microglial clusters displayed enhanced expression of interferon-stimulated genes and repair-associated programs (Fig. 2E and F).

These transcriptomic changes were accompanied by morphological alterations in repopulated microglia, including decreased cell and soma circularity and higher spatial complexity, indicative of a less amoeboid and more ramified morphology (Supplementary Fig. 3). Together, these observations suggest that repopulated microglia exhibit transcriptional and morphological features consistent with altered reactivity and with gene programmes associated with tissue repair and neuroprotection, particularly within the homeostatic compartment, relative to the vehicle group. Because samples from four mice were pooled within each treatment group, the analysis resolved microglial heterogeneity at the cellular level but did not preserve the biological replication needed to assess inter-animal variability; these findings should therefore be interpreted as descriptive and hypothesis-generating.

### Short-term microglia depletion accelerates fine motor recovery after stroke

**Figure 3:**
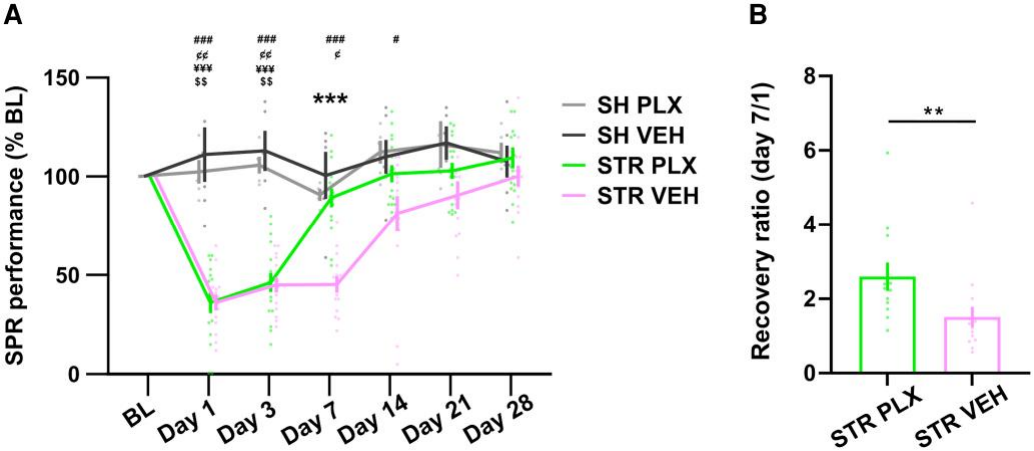
**Short-term microglia depletion after stroke accelerates motor recovery in SPR task.** (**A**) SPR performance relative to baseline (%). STR PLX_1_: *n* = 16, STR VEH_2_: *n* = 14, SH PLX_3_: *n* = 6, SH VEH_4_: *n* = 5. Mixed-effects model (REML) with Tukey’s multiple comparisons test. *** *P* < 0.001 versus groups 1 and 2; $$ *P* < 0.01 versus group 1 and 3; ¥¥¥ *P* < 0.001 versus group 1 and 4; ¢ *P* < 0.05 versus group 2 and 3; ### *P* < 0.001 versus group 2 and 4. (**B**) Recovery ratio (day7/1) direct comparison between the stroke groups. STR PLX: *n* = 12, STR VEH: *n* = 14. Mann–Whitney test. *n* reflects animals with available paired measurements. 1 = no change; > 1 = improvement. ** *P* < 0.01; *, $, ¥, ¢, # *P* < 0.05 Data are shown ± SEM. Each data point represents a single mouse. BL, baseline; PLX, PLX5622; SH, sham; STR, stroke; VEH, vehicle.

To assess motor recovery, we chose the dMCAo model as it affects fine motor function that can be evaluated using the SPR task (Fig. 3A). Paw preference was determined during a training phase and was comparably distributed across groups: stroke PLX-treated (STR PLX: 8 right, 8 left); stroke vehicle-treated (STR VEH: 8 right, 7 left); sham PLX-treated (SH PLX: 1 right, 8 left); sham vehicle-treated (SH VEH: 2 right, 7 left). At the time of intervention, animals of all groups did not differ significantly in weight (SH VEH 25.34 ± 0.60 g, SH PLX 25.44 ± 0.68 g, STR VEH 25.33 ± 0.61 g, STR PLX 25.34 ± 0.49 g; one-way ANOVA, *P* = 0.997).

Consistent with prior work,^20^ dMCAo induced a marked drop in SPR performance on day 1 in comparison to shams (STR PLX and STR VEH versus SH PLX: *P* = 0.007 and *P* = 0.009, respectively; versus SH VEH: both *P* < 0.001), with no differences between stroke groups (*P* > 0.999). On day 7, stroke PLX-treated animals showed a drastically improved performance, nearly reaching baseline levels and no longer differing significantly from sham animals (SH PLX: *P* > 0.814; SH VEH: *P* = 0.994), while the stroke vehicle-treated animals were still showing significant impairments (SH PLX: *P* = 0.026; SH VEH: *P* < 0.001). In direct comparison, PLX-treated animals after stroke exhibited a 1.8-fold greater recovery between day 1 and day 7 than vehicle-treated mice after stroke, with median recovery rates of 2.255 versus 1.275, *P* = 0.004 (Fig. 3B).

On day 14, PLX-treated stroke animals showed a sustained functional improvement (% SPR baseline) while vehicle-treated stroke animals still demonstrated slight impairments (% SPR baseline, SH VEH, *P* = 0.043) which did not reach statistical significance in comparison to PLX-treated animals (SH PLX, *P* = 0.139; STR PLX, *P* = 0.213). From day 21 onward, no differences were observed between any groups (all *P* ≥ 0.140).

Throughout the entire study, no differences were detected between both sham groups (day 1: *P* = 0.934; day 3: *P* = 0.912; day 7: *P* = 0.851; day 14: *P* = 0.995; day 21: *P* > 0.999; day 28: *P* = 0.968), confirming that PLX alone did not affect task performance in uninjured animals.

Spontaneous behaviour assessed in the open field did not differ significantly between the two stroke groups. Open field test showed comparable locomotor activity, exploration, and movement patterns across conditions, and no differences in weight were observed throughout the study indicating that general food intake was not significantly hampered by fine motor deficits (Supplementary Fig. 4).

In summary, timed microglia depletion led to a faster recovery of fine motor function of the contralateral forepaw. Moreover, this functional improvement remained stable during the repopulation of microglia and persisted until microglia counts reached normal values.

### Increased connectivity within ipsilesional M1 informs functional recovery

**Figure 4:**
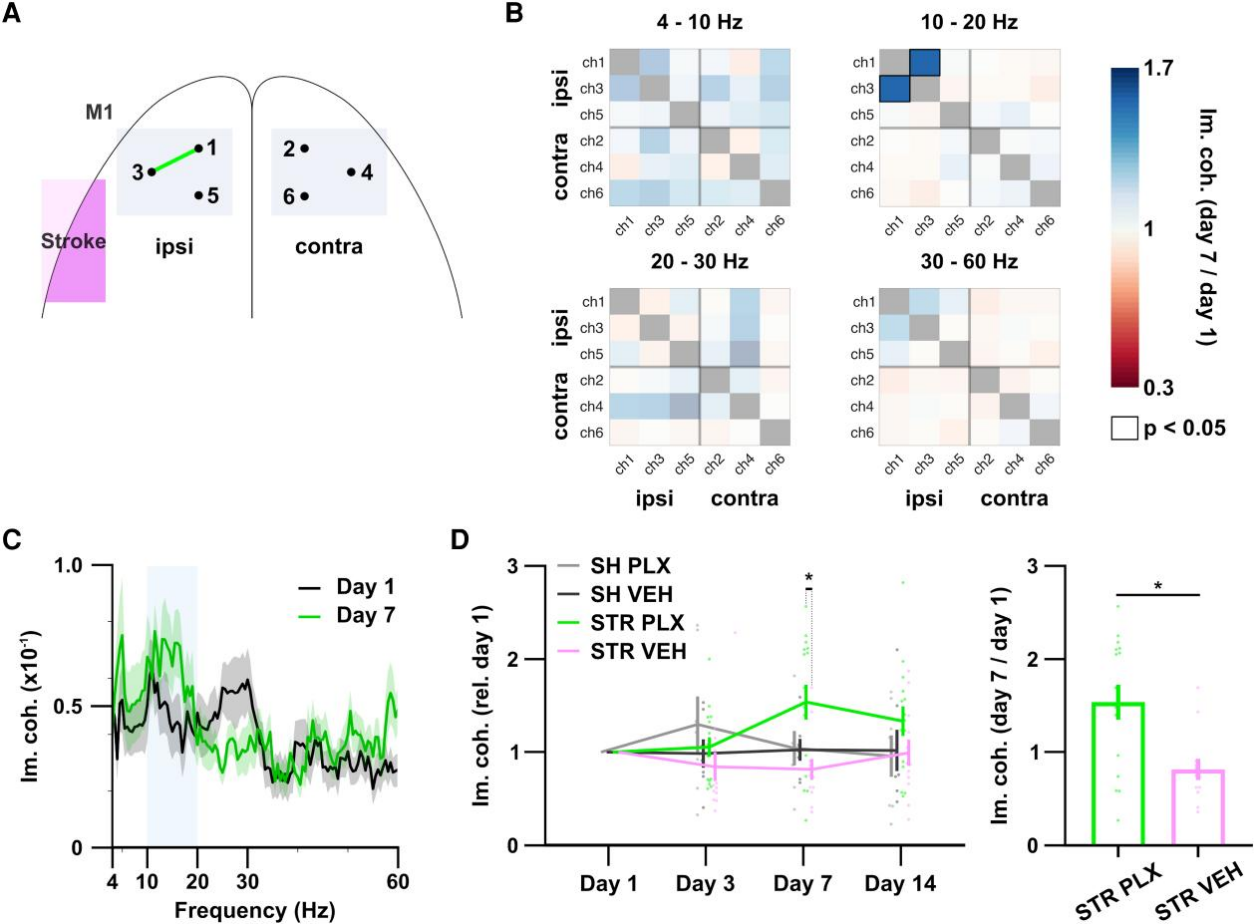
**Increase of 10–20 Hz connectivity (imaginary coherence) in ipsilesional M1 of PLX-treated mice.** (**A**) Schematic illustrating electrode location within M1, line indicates a significant increase of imaginary coherence compared with the vehicle-treated stroke group. (**B**) Heat maps depicting increases and decreases of imaginary coherence on day 7 in relation to day 1 in given frequency bands for all possible electrode combinations. Significant alterations compared with the vehicle-treated stroke group are outlined. SH PLX: *n* = 8; SH VEH: *n* = 7; STR PLX: *n* = 14; STR VEH: *n* = 12. Mixed-effects model (REML) with Tukey’s multiple comparisons test. (**C**) ch1-ch3 imaginary coherence spectrum of PLX-treated mice on day 1 and day 7 after stroke. STR PLX: *n* = 14. (**D**) Left, the fold-change of ch1-ch3 10–20 Hz imaginary coherence in all four groups in relation to day 1. SH PLX: *n* = 7; SH VEH: *n* = 7; STR PLX: *n* = 15; STR VEH: *n* = 12. Mixed-effects model (REML) with Tukey’s multiple comparisons test. Right, direct comparison of the evolution of imaginary coherence in the two stroke groups. STR PLX: *n* = 15; STR VEH: *n* = 12. Mann–Whitney test. 1 = no change; >1 = increase relative to day 1. Each data point represents a single mouse. * *P* < 0.05. Data are shown ± SEM. im. coh., imaginary coherence; PLX, PLX5622; rel., relative to; SH, sham; STR, stroke; VEH, vehicle.

To investigate the underlying neurophysiological processes associated with enhanced regenerative capacity, we recorded ECoG signals from both hemispheres, covering extensive areas of the motor and sensory cortices (Fig. 4A). In our previous work,^20^ we had identified the area covered by electrode ch3, within the ipsilesional primary motor cortex (M1), as a key region involved in recovery. Given the multifaceted role of microglia in modulating synaptic turnover and receptor composition, we hypothesized that microglial depletion might result in significant alterations in functional network connectivity.

To assess connectivity, we calculated imaginary coherence during the recovery phase spanning from day 1 to day 7 post-stroke.^31^ For spectral and connectivity analysis, we defined frequency bands as follows: theta (4–10 Hz), low beta (10–20 Hz), high beta (20–30 Hz), and gamma (30–60 Hz).

First, we examined the connectivity between all possible electrode connections over ipsilesional and contralesional M1 during active episodes, defined by locomotion and exploratory behaviour (Fig. 4A). Connectivity in the lower beta band was significantly increased, by up to ∼150%, in the post-stroke-PLX-treated group compared with vehicle-treated stroke animals. This increase was only evident between electrodes ch1 and ch3 in the ipsilesional M1 on day 7 (mean difference 0.723, *P* = 0.017, mixed-effects model (REML); Fig. 4B-D) and was observed only during active, not resting, periods (day 7: mean diff. 0.155, *P* = 0.969, mixed-effects model (REML); Supplementary Fig. 5D). Supporting analysis showed that the increase in beta band connectivity spanned a broadband frequency range between 10 and 20 Hz and did not coincide with the ∼16 Hz peak observed in the power spectrum (PSD mean 15.63 ± 0.19 Hz versus im. coh. mean 13.49 ± 0.39 Hz, *P* < 0.001, Welch’s *t*-test; Fig. 4C, Supplementary Fig. 5A, B). Analysis of the aperiodic exponent of the power spectrum in M1, a marker of cortical excitation-inhibition balance, did not reveal significant differences between PLX-treated and vehicle-treated post-stroke animals (Supplementary Fig. 5E). Furthermore, the observed increase in beta connectivity was neither correlated with spectral power in the corresponding frequency band nor with aperiodic markers (Supplementary Table 5).

**Figure 5:**
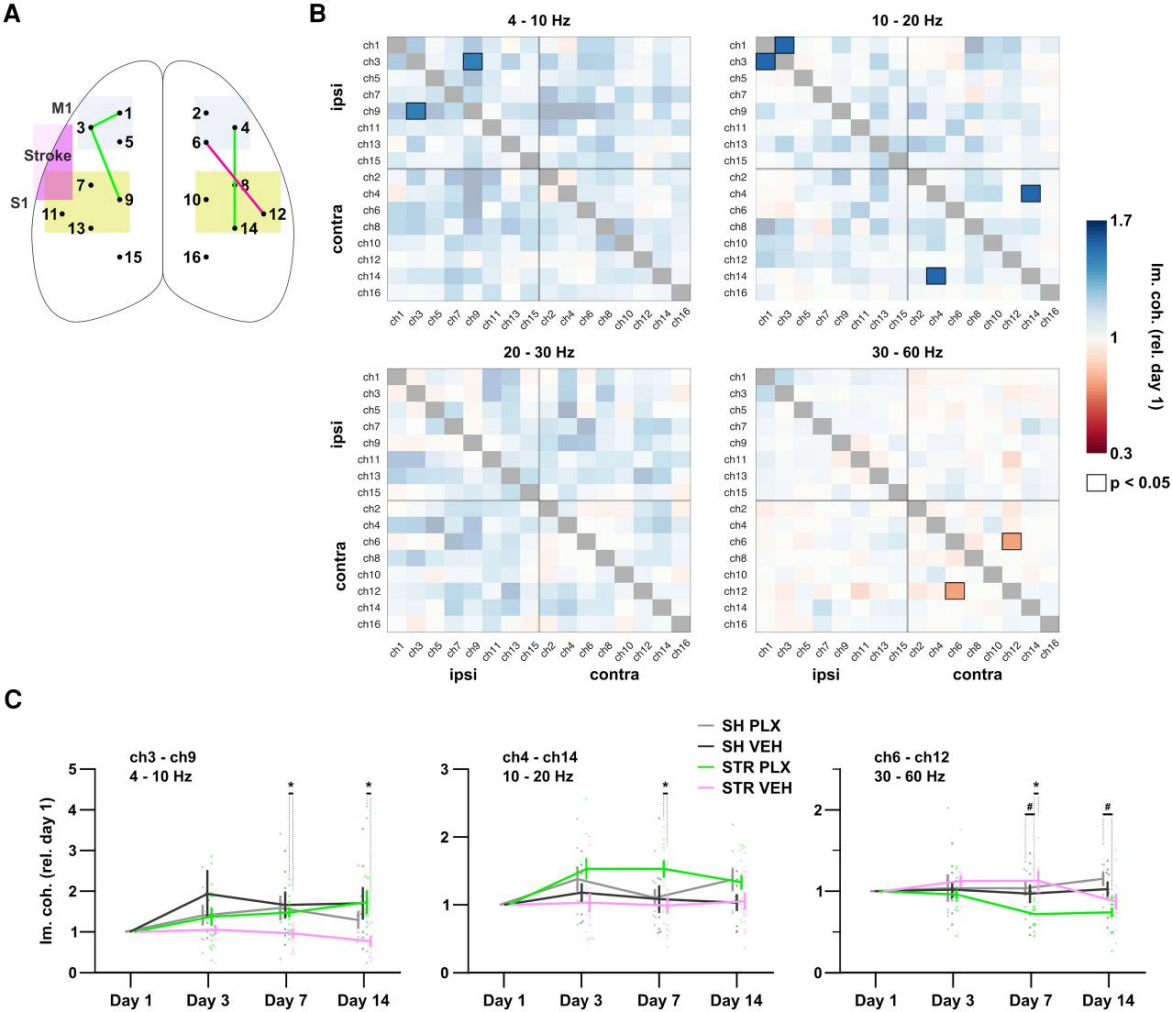
**Bilateral modifications of sensorimotor networks following ischaemic stroke in PLX-treated mice.** (**A**) Schematic illustrating all electrode locations, green line indicates significant increases and red line significant decreases of imaginary coherence (im. coh.) compared with the vehicle-treated stroke group. (**B**) Heat maps depicting increases and decreases of imaginary coherence on day 7 in relation to day 1 in given frequency bands for all possible electrode combinations (color-coded). Significant alterations compared with the vehicle-treated stroke group are outlined. SH PLX: *n* = 8; SH VEH: *n* = 7; STR PLX: *n* = 14; STR VEH: *n* = 12. Mixed-effects model (REML) with Tukey’s multiple comparisons test. (**C**) The fold-changes of imaginary coherence in the given frequency bands and between given electrode combinations in all four groups in relation to day 1 are demonstrated. Sample sizes varied depending on electrode combinations: Theta (ch3–ch9): SH PLX *n* = 8; SH VEH *n* = 7; STR PLX *n* = 15; STR VEH *n* = 12. Beta (ch4–ch14): SH PLX *n* = 8; SH VEH *n* = 8; STR PLX *n* = 15; STR VEH *n* = 14. Gamma (ch6–ch12): SH PLX *n* = 8; SH VEH *n* = 9; STR PLX *n* = 15; STR VEH *n* = 11. Mixed-effects model (REML) with Tukey’s multiple comparisons test. Each data point represents a single mouse. *, # *P* < 0.05. Data are shown ± SEM. im. coh., imaginary coherence; PLX, PLX5622; SH, sham; STR, stroke; VEH, vehicle; rel., relative to.

Next, we assessed intrahemispheric connectivity between the motor and sensory network (Fig. 5A and B). In PLX-treated animals, a significant increase in theta connectivity between ipsilesional motor and sensory cortices (ch3–ch9) was observed when compared with vehicle-treated stroke animals (day 7: mean diff. 0.513, *P* = 0.043, day 14: mean diff. 0.960, *P* = 0.039, mixed-effects model; Fig. 5B). This effect appeared to be primarily driven by a decrease in connectivity within the vehicle-treated stroke animals over time (Fig. 5C). On the contralateral side, we found an increase in lower beta connectivity (ch4–ch14) beginning as early as day 3 (day 7: mean diff. 0.540, *P* = 0.28, mixed-effects model; Fig. 5B and C), and a significant decrease in gamma connectivity (ch6–ch12) emerging by day 7 (STR PLX versus STR VEH day 7: mean diff. −0.410, *P* = 0.031, SH PLX versus STR PLX day 7: mean diff. 0.318, *P* = 0.032, day 14: mean diff. 0.412, *P* = 0.011, mixed-effects model; Fig. 5B and C). No significant changes were detected in the upper beta frequency band (Fig. 5B). While the ipsilesional increase in theta connectivity correlated with theta power in the motor cortex (ch3 4–10 Hz power versus ch3–ch9 4–10 Hz im. coh., *r* = −0.434, *P* = 0.019; repeated-measures correlation), no such relationship was found for other channel pairs (Supplementary Table 5).

**Figure 6:**
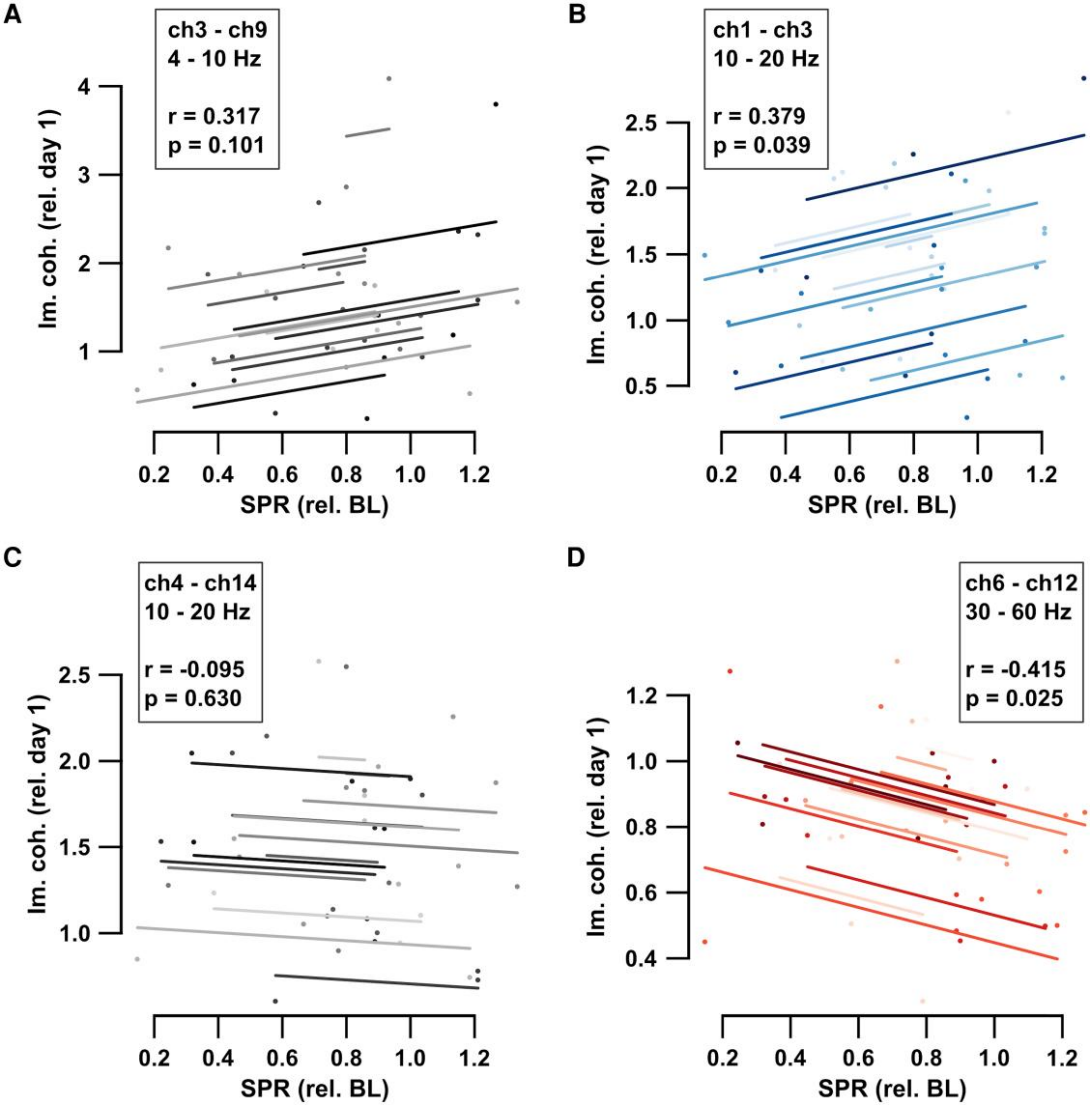
**Lower beta connectivity within ipsilesional M1 of PLX-treated mice correlates with behavioural recovery.** (**A–D**) Repeated-measures correlations of imaginary coherence (in relation to day 1) between given electrode pairs in given frequency bands and behavioural performance (in relation to baseline, BL) are shown for the recovery phase (day 3, day 7 and day 14) in PLX-treated mice after ischaemic stroke. B indicates a significant positive correlation, D indicates a significant negative correlation. *n* = 14. Each data point represents a single mouse at a given timepoint. BL, baseline; im. coh., imaginary coherence; rel., relative to; SPR, single-pellet reaching.

To assess whether these observations relate to improvements in fine motor skills, we examined the link between functional connectivity and motor recovery using repeated-measures correlation during the recovery phase (day 3 to day 14). M1 ipsilesional connectivity in the low beta range positively correlated with performance in the SPR task, supporting a potential mechanistic link between local network reorganization and functional recovery (*r*= 0.379, *P* = 0.039, repeated-measures correlation; Fig. 6B). The behavioural correlation was specific to M1 and was not found for ipsi- or contralesional sensorimotor connectivity in the theta and low beta range respectively (Fig. 6A and C). Conversely, contralesional sensorimotor gamma connectivity was significantly and inversely correlated with fine motor improvement (*r* = −0.415, *P* = 0.025, repeated-measures correlation; Fig. 6D).

In summary, we identified bilateral alterations in sensorimotor network dynamics following stroke. Among these, the increase in low beta-band connectivity within the ipsilesional motor cortex emerged as a strong candidate mechanism underlying the restoration of contralateral forepaw function. Additionally, reduced gamma synchronization in the contralateral sensorimotor system was likewise associated with fine motor recovery.

## Discussion

Here we show for the first time that short-term microglia depletion between days 3 and 7 after sensory cortical stroke in mice accelerates fine motor recovery and modulates functional connectivity within bilateral sensorimotor networks. While the depletion itself did not affect lesion size, spontaneous behaviour, or general locomotion, it led to a significant early improvement in skilled forelimb use. This early recovery coincided with the peak of depletion, correlated with increased connectivity within the ipsilesional motor cortex and persisted throughout microglial repopulation, suggesting that microglial activity during this subacute phase critically shapes the trajectory of post-stroke recovery.

Microglia is rapidly activated in response to cellular injury. In the immediate aftermath of ischaemic stroke, microglia accumulate at the lesion site, where they release pro-inflammatory mediators that sustain neuroinflammation and can contribute to secondary neuronal damage.^4^ Simultaneously, they play a beneficial role by clearing necrotic tissue and secreting anti-inflammatory markers and growth factors, thereby supporting vascular remodelling and tissue repair.^28^ This dual role highlights the temporally and spatially heterogeneous impact of microglia on post-stroke recovery.^3,5^ Consequently, therapeutic strategies targeting microglial activation states or phenotypes are of increasing interest. Microglia depletion prior to ischaemia consistently leads to worse outcomes, including larger infarcts and impaired functional recovery, underscoring their protective function during the acute phase.^32-35^ Similarly, sustained depletion from the acute through the chronic phase leads to detrimental behavioural outcomes.^7^ In contrast, transient microglia depletion limited to the subacute phase—particularly between days 3 and 7 post-stroke—has emerged as a promising approach.^19^ This intervention has been shown to reduce neuroinflammation, enhance recovery, and shift microglial gene expression profiles toward a more homeostatic phenotype following repopulation.^6,17-19^ In line with these findings, our data demonstrate that timed microglia depletion between days 3 and 7 after sensory cortical stroke leads to improved performance in the SPR task by day 7 post-stroke, corresponding to the peak of depletion, whereas vehicle-treated controls required 14 to 21 days to reach similar functional levels. Notably, the behavioural improvement persisted through microglial repopulation, reaching day 28 post-stroke, when the microglial population was fully restored. These findings suggest that short-term disruption of microglial activity during a defined subacute period can initiate an accelerated and enduring recovery process. Consistent with existing literature, activated residual microglia may inhibit recovery while newly repopulated microglia facilitate tissue repair.^36^ Our scRNA-seq analyses suggest that repopulated microglia exhibit altered expression of genes associated with repair, DAM-related states, and inflammatory responses. However, these changes do not recapitulate full canonical DAM or previously described PLX-repopulation transcriptional programs, indicating a partial and context-dependent transcriptional shift rather than a distinct plasticity-associated program.^19,28^ The precise molecular pathways mediating these effects remain to be determined.

Microglial activation states influence neuronal network dynamics. Both short- and long-term microglial depletion impact synaptic structure and function.^10,37^ Chronic depletion results in increased dendritic spine density and synaptic contacts.^6,15,37^ In the visual cortex, microglial depletion led to a transient increase in both excitatory and inhibitory synapses on excitatory neurons, accompanied by heightened activity of both excitatory and inhibitory cell populations, and these changes were reversible following repopulation.^38^ At the synaptic level, tumour necrosis factor-alpha (TNF-α) released by activated microglia modulates synaptic receptor trafficking through endocytotic and exocytotic mechanisms.^2,39^ Activated microglia have also been shown to slow gamma oscillations in situ, although the underlying mechanisms remain incompletely understood.^2,13^ Behaviourally, microglial depletion has been associated with enhanced spatial memory and improved recovery following hippocampal injury.^6,15,37,40^ Collectively, these findings indicate that microglial depletion induces structural and functional changes that influence cortical information processing and motor and cognitive performance. The rapid restoration of contralateral skilled motor function and the observed modifications of bilateral sensorimotor networks suggest that targeted suppression of activated microglia during a critical subacute window after ischaemic injury may facilitate synaptic plasticity and network reorganization, thereby enhancing functional recovery.

Several human studies have linked cortical reorganization via functional connectivity and behavioural outcome following ischaemic stroke.^41-43^ From a neuroanatomical and systems-level perspective, the observed increase in functional connectivity within the ipsilesional motor cortex in our study is noteworthy, as it was significantly associated with improved contralateral motor performance. This region comprises not only the primary motor cortex (M1; channel 3) but also the transition zone between the primary and secondary motor cortices (channel 1, see also Supplementary Table 5). Accordingly, this finding is consistent with human data demonstrating beneficial effects of enhanced connectivity between primary and secondary, more specifically, supplementary motor areas in stroke recovery.^44-47^ In our data, the frequency range in which this strengthened coupling occurred corresponds to the lower beta band (10–20 Hz). Beta-band activity is classically associated with the sensorimotor cortex and basal ganglia and is typically defined in the 13–30 Hz range.^48,49^ However, recent studies have demonstrated the functional relevance of striatal 10 Hz oscillations for locomotion in mice, and growing evidence from rodent, macaque and human supports a subdivision into low (<20 Hz) and high (>20 Hz) beta components.^50-54^ Computational analyses suggest that low beta rhythms exhibit favorable properties, such as the ability to link neuronal ensembles that are otherwise segregated by gamma oscillations.^55^ Moreover, beta synchrony around 20 Hz between M1 and somatosensory regions has been implicated in sensorimotor integration in macaques.^56^ Interestingly, the observed increase in motor cortical connectivity was not associated with changes in beta power nor with aperiodic markers previously linked to the cortical excitation–inhibition (E/I) balance.^20,57^ While an increase in aperiodic exponents at channel 3 was associated with deficits in fine motor performance in a previous study, no significant differences in this parameter were observed between the PLX-treated and vehicle-treated groups in the present dataset.^20^ This may suggest that mechanisms beyond a mere shift in cortical E/I balance contributed to the observed recovery. In addition, our data indicate a significant decrease in connectivity within a contralesional sensorimotor network, which temporally coincided with the early recovery of fine motor skills in the forepaw ipsilateral to this network. The neuroanatomical interpretation of this finding is less straightforward. Since ECoG recordings were acquired during free movement, it may be speculated that the early restoration of motor function influenced compensatory recruitment of contralateral networks or led to altered forelimb usage. Further studies will be required to elucidate this finding, as well as the specific roles of the two additional ipsilesional and contralesional network modifications described above. Taken together, the observed increase in low beta connectivity (<20 Hz) within the ipsilesional motor cortex may reflect the formation of expanded motor networks involving secondary motor areas and could represent a compensatory mechanism facilitating recovery.

The findings should be interpreted in light of the study’s limitations. It remains uncertain whether repopulated microglia fully recapitulates the functional properties of their predecessors, and the present data do not resolve the cellular interactions between microglia and neurons that facilitate the emergence of coherent and functionally integrated cell assemblies during recovery. Future studies incorporating transcriptomic profiling and selective manipulation of microglial subsets will be necessary to delineate the underlying pathways. Methodologically, longitudinal MRI assessment was performed in a small cohort and relied on T2-weighted imaging, which may limit sensitivity to subtle changes in lesion evolution and peri-infarct tissue dynamics. In addition, the exclusive use of male animals and the reliance on a single behavioural assay may constrain generalizability and the breadth of functional assessment. Finally, due to technical constraints, ECoG recordings were acquired outside task performance and were of relatively short duration. Therefore, whether and how cortical spreading depolarizations, which have been shown to contribute to secondary injury after stroke, are dynamically influenced by microglia and affect functional connectivity remains to be investigated in future studies.^58-60^

Taken together, our findings underscore the critical role of microglia in shaping post-stroke recovery trajectories and highlight their potential as a therapeutic target through temporally controlled modulation of their activity.

## Supplementary Material

fcag202_Supplementary_Data

## Data Availability

The data that support the findings of this study and all custom-written MATLAB codes are provided in the Supplementary Material.
