# Supplementary material for: Timed microglia depletion promotes functional network reorganization and motor recovery after stroke
Source: Brain Commun. 2026 Jun 2;8(3):fcag202. doi: 10.1093/braincomms/fcag202 (PMC13256948; doi:10.1093/braincomms/fcag202)
Supplement: fcag202_Supplementary_Data [file fcag202_supplementary_data.zip › Supplementary_Materials.pdf]

Supplementary Material

Timed microglia depletion promotes functional network reorganization and motor recovery after stroke

Sara Isla Cainzos[1*], Fanny Quandt[1], Malte Borggrewe[2], Hanna-Marie Altjohann[1], Tim Magnus †[1] and Jonatan Biskamp†[1]

† These authors contributed equally as senior authors.

Author affiliations:

1 Department of Neurology, University Medical Center Hamburg-Eppendorf, 20246, Hamburg, Germany

2 Independent Bioinformatics Consultant, 20259, Hamburg, Germany

Correspondence to: Sara Isla Cainzos

Department of Neurology, University Medical Center Hamburg-Eppendorf, 20246, Hamburg, Germany

E-mail: s.islacainzos@uke.de

Content:

Expanded Materials and Methods

Supplementary Figures 1-5

Supplementary Tables 1 & 5 (included in Supplementary PDF)

Supplementary Tables 2-4 (provided as Excel files)

Custom-written MATLAB code (provided as .zip archive)

Expanded Materials and Methods

Ethics statement

C57Bl/6 male mice aged nine postnatal weeks at the start of the experiment provided by the animal facility of the University Medical Center Hamburg-Eppendorf were used in this study. Experimental protocols were approved by the Behörde für Justiz und Verbraucherschutz der Freien und Hansestadt Hamburg (approval numbers N53/2020 and N102/2023). All procedures followed the guidelines of the animal facility of the University Medical Center Hamburg-Eppendorf and complied with the Guide for the Care and Use of Laboratory Animals. Animals were housed in groups under a 12h dark-light cycle. After implantation of electrodes mice were housed individually for 49 days.

Study design

All animals underwent identical surgical procedures and behavioral tests except for stroke versus sham intervention. Group sizes were chosen based on previous studies and the expected variability in behavioral and electrophysiological measures. Animals were randomly assigned to experimental groups. Intervention surgeries and behavior were performed by the same experimenter. During the execution of behavioral experiments, the experimenter was blinded to group allocation, group allocation could not be derived from cage location or animal number. Electrophysiological preprocessing and connectivity analyses, as well as histological quantification and flow cytometry data analysis, were conducted by investigators blinded to surgery and treatment.

For the first cohort, a total of 24 mice were used, no animals were excluded from the analysis (Fig. 1A).
For the second cohort, according to our approved protocol, animals that neither contributed to behavioral ("non-learners") nor electrophysiological (artifactual ECoG recordings) measures were immediately excluded from the study (n=9). Two animals died after surgical interventions. Taken together, 49 animals underwent electrode implantation and 41 were included in the final analysis. From these animals 42 were trained in the SPR task and one was excluded as a "non-learner", 41 animals were included in the analysis. The 7 extra animals were included in the experiment to complete the electrophysiological analysis to replace the exclusions for bad recordings, thus they were not trained in the SPR task. Open Field was performed in all the 49 animals (Fig. 1A).

For the third cohort, a total of 8 animals were used, no animals were excluded from the analysis.

Electrode implantation and dMCAo surgery

Mice were chronically implanted with epidural ECoG electrodes as previously described[1]. Briefly, animals received buprenorphine (0.05 mg/kg, subcutaneous) prior to the surgery and were anesthetized with isoflurane (3–5% induction, 1–2% maintenance in O_2). Body temperature was maintained with a heating pad. 16 small craniotomies were performed, and platinum/iridium wire electrodes were positioned epidurally over both hemispheres with the dura left intact. A cerebellar reference and skull screw ground were used. Electrodes were secured with dental cement and connected to a connector. Exact stereotaxic coordinates are described in detail in Supplementary Table 1.

Ischemic stroke was induced using a permanent distal middle cerebral artery occlusion (dMCAo) model without reperfusion**,** as previously described[1]. Under isoflurane anesthesia, a small skin incision was made between the eye and ear, the temporal muscle was retracted, and a craniotomy was performed over the MCA. The artery was permanently coagulated at its distal bifurcation. If no clear bifurcation was visible, the proximal distal MCA segment was coagulated. Stroke was induced contralateral to the dominant forepaw, previously determined; if no preference was observed, the hemisphere was randomly assigned. Sham-operated animals underwent identical procedures without arterial coagulation. Postoperatively, animals received tramadol in drinking water and softened food for 72 hours. A recovery period of one week was allowed after electrode implantation before behavioral training or electrophysiological recordings began.

Behavior

All behavioral experiments were performed in a dedicated testing chamber under constant illumination and were video recorded for offline verification. Mice were habituated to the room for at least 30 minutes prior to testing. Apparatuses were cleaned with 70% ethanol between animals. Testing was performed at fixed times of day. The single-pellet reaching task was conducted in the morning and open field testing was performed in the evening. Sessions were separated by an interval of at least 6 hours, allowing a sufficient recovery before the evening behavior.

Open Field

General locomotor activity was assessed in an open field arena (60 cm diameter). Mice freely explored the arena for 12 minutes while being recorded with an overhead camera; electrophysiological data was recorded at the same time using a wireless system. Locomotor parameters were analyzed offline using ANY-maze Video Tracking System 7.62 Software (Stoelting Co.).

Single-pellet reaching task (SPR)

Fine motor skills were evaluated using the single-pellet reaching task as previously described[1]. Mice were placed in a transparent chamber and trained to reach with their preference paw through a window to retrieve a food pellet placed outside and eat it. In our protocol millet seeds served as food pellets. Training began one week after electrode implantation and continued for a maximum of 14 consecutive days. Each training session lasted up to 20 minutes or 30 reaching attempts. During the first week, forepaw preference was determined (>65% use within one session). Subsequently, millet seeds were presented exclusively on the preferred side and individual baselines were defined by averaging the number of successful reaching attempts on the last 2 days of training. An attempt was scored as successful if the millet seed was grasped and consumed without falling from the mouse paw in between. Animals failing to reach predefined learning criteria (≥10 successful reaches out of 30 attempts after 7 days) were classified as non-learners and excluded from SPR analysis. Testing sessions after intervention were performed accordingly and performance was assessed relative to baseline (in %). Trials with fewer than 30 attempts within 20 minutes were classified as "did not perform" and excluded from analysis. Mice were food-restricted overnight prior to testing sessions (10-12 hours' maximum) to maintain task engagement, and food was returned afterwards.

References

1. Biskamp J, Isla Cainzos S, Higgen LF, Gerloff C, Magnus T. Normalization of aperiodic electrocorticography components indicates fine motor recovery after sensory cortical stroke in mice. *Stroke*. 2022;53(9):2945-2953. doi:10.1161/strokeaha.122.039335

Supplementary Figures

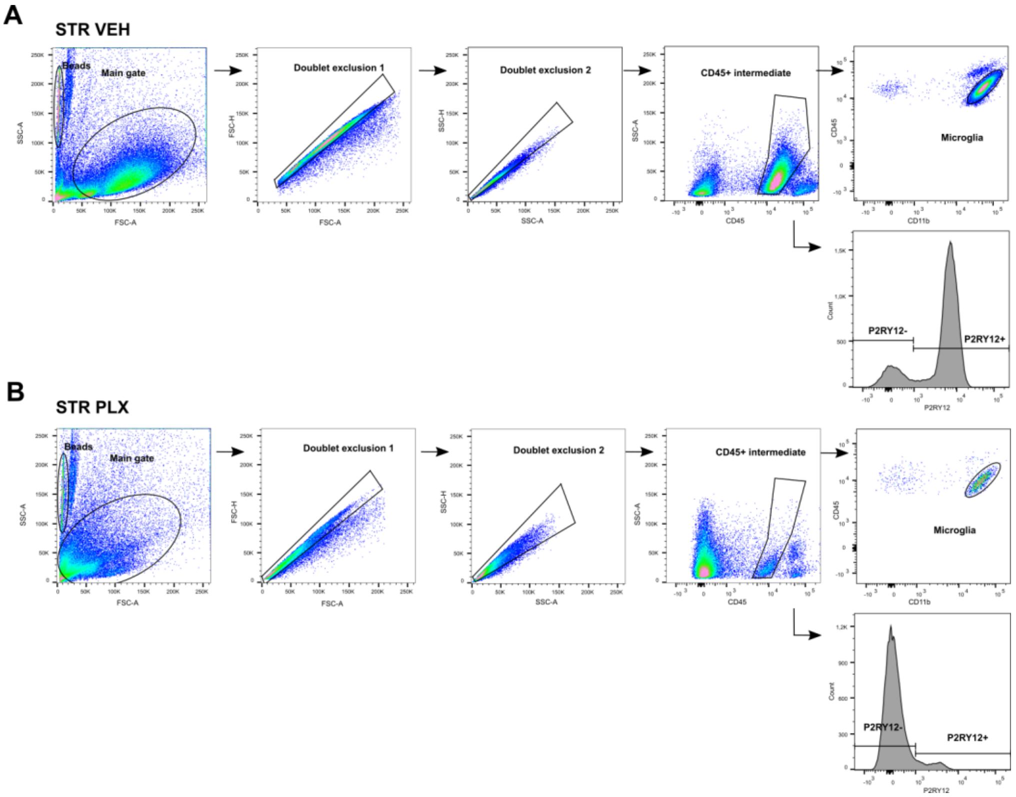

Supplementary Figure 1. Flow cytometry gating strategy for defining microglial cell populations. A, Representative flow cytometry analysis plots from STR VEH showing sequential gates: main singlet population, bead exclusion, doublet exclusion 1 and 2, CD45^int^ cells, and final microglia gate defined as CD45^int^CD11b^+^P2RY12^+^ microglia, with P2RY12 used to exclude P2RY12^-^ infiltrating cells. **B,** Identical gating applied to STR PLX illustrating near-complete microglial depletion. SH, sham; STR, stroke; PLX, PLX5622; VEH, vehicle; P2RY12, purinergic receptor P2Y12.

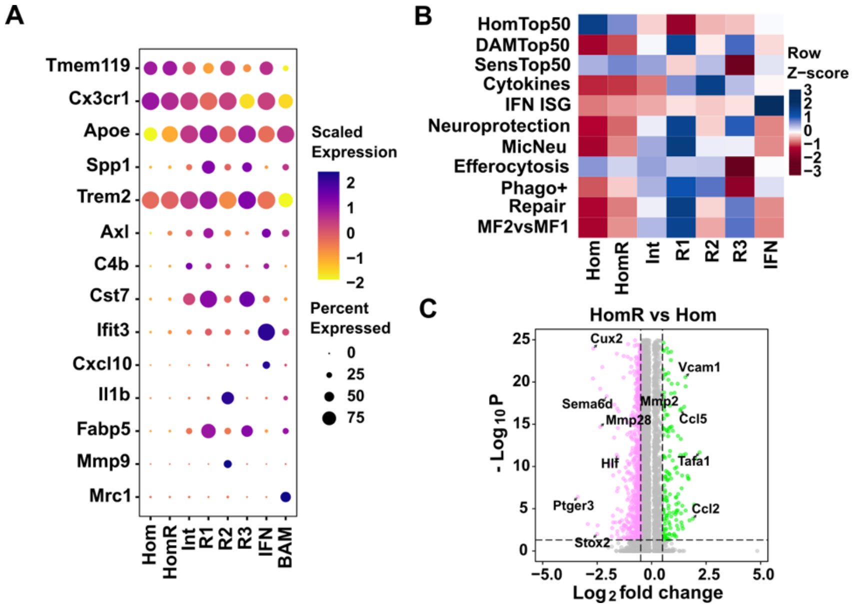

Supplementary Figure 2. Supporting analyses of repopulated microglia at day 14 post-stroke. A, Dot plot of annotated marker genes used for UMAP-based cluster classification. **B,** Heatmap of gene set activity (GSA) scores across clusters. STR PLX: 10,378 cells; STR VEH: 10,744 cells (pooled from n=4 mice per group). **C,** Volcano plot of differentially expressed genes (DEGs) between selected clusters (log2FC > 0.5, p < 0.05). STR, stroke; PLX, PLX5622; VEH, vehicle; Hom, homeostatic; HomR, homeostatic reactive; Int, intermediate; R1–R3, reactive subtypes 1–3; IFN, interferon-responsive; BAM, border-associated macrophages; HomTop50, homeostatic top 50 genes; DAMTop50, disease-associated microglia top 50 genes; SensTop50, sensome top 50 genes; ISG, interferon-stimulated genes; MicNeu, microglia-neuron interaction; Phago+, positive regulation of phagocytosis; MF1/MF2, macrophage subtype 1 and 2.

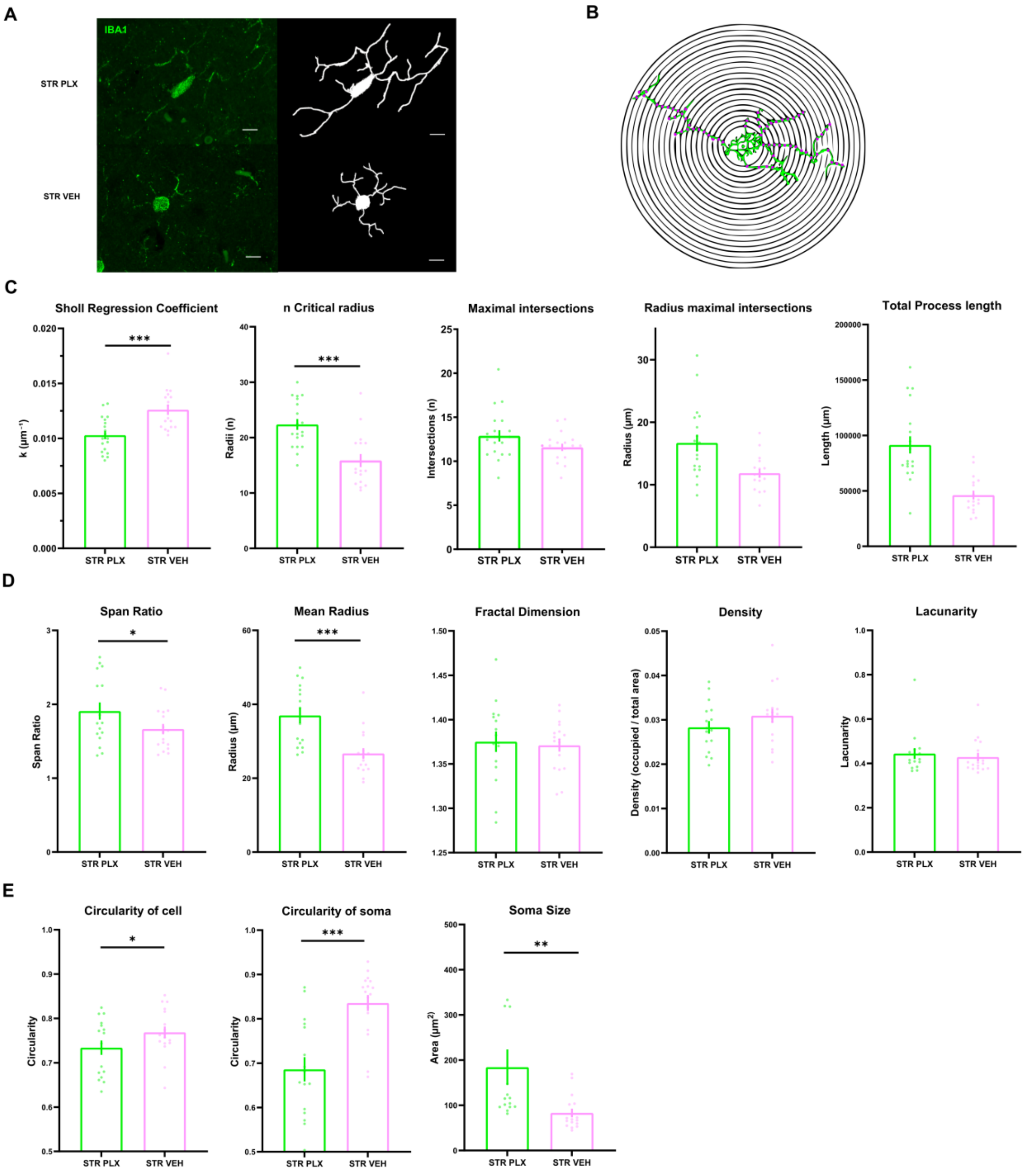

Supplementary Figure 3. Morphological changes in microglia following one week of repopulation after PLX-treatment at day 14 post-stroke. A, Left, representative IBA1-stained microglia from STR PLX and STR VEH groups. Right, corresponding fractal analysis illustrations of the same cells. **B,** Representative image of Sholl analysis. **C,** Sholl analysis results showing increased number of critical radii and decreased Sholl regression coefficient in STR PLX vs STR

VEH (***p<0.001); no significant differences in total process length, radius of maximal intersections, or maximal intersections. **D,** Fractal analysis showing increased span ratio (*p=0.014) and mean radius (***p<0.001); fractal dimension, lacunarity, and density were not significantly different. **E,** Morphometric parameters showing increased soma size (**p=0.002), decreased cell circularity (*p=0.029), and reduced soma circularity (***p<0.001) in STR PLX vs STR VEH. **For panels C–E**, STR PLX: n = 3; STR VEH: n = 3. Each datapoint represents a single microglial cell; 10–14 cells were analyzed per mouse. Statistical analyses were performed using nested t-tests or Mann–Whitney tests as appropriate. White scale bar in A indicates 10 µm. *p<0.05, **p<0.01, ***p<0.001. Data are shown ± SEM. STR PLX, stroke PLX5622; STR VEH, stroke vehicle. Note: Sholl and fractal analysis were performed on 5 µm sections. Fine microglial processes extending out of the plane of section are not captured.

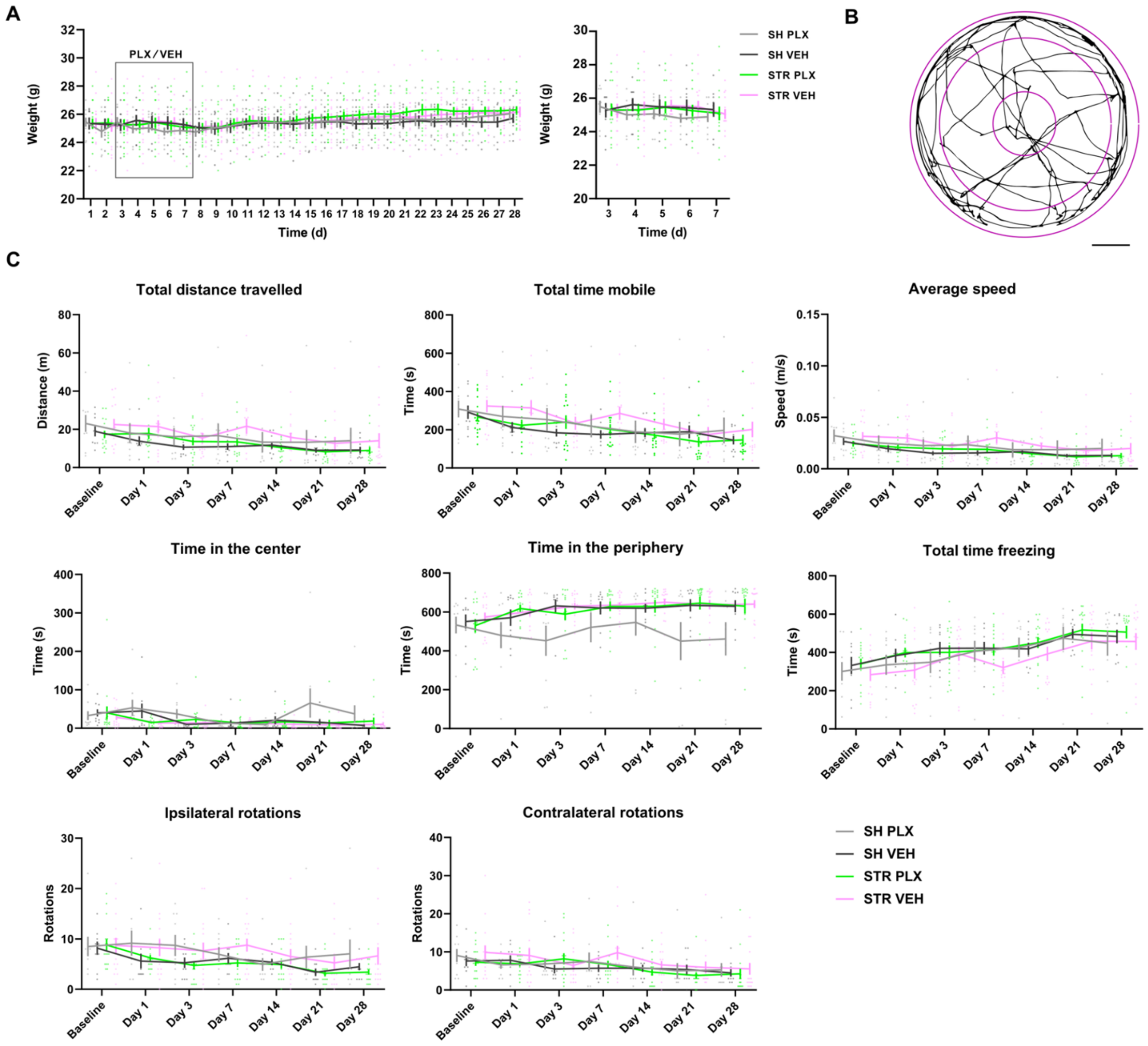

Supplementary Figure 4. Body weight and open field behavior over time after stroke. A, Left, Body weight across the experiment after stroke or sham intervention (d=0). Right, expanded view of days 3–7 post-surgery, corresponding to the PLX5622 treatment window. **B,** Schematic of an open field arena recording of 12 minutes, purple lines illustrating periphery, intermediate and center zones. **C,** Plots illustrating open field indicated measures. SH PLX: n = 9; SH VEH: n = 9; STR PLX: n = 16; STR VEH: n = 15. Black scale bar in B indicates 10 cm. Data are shown ± SEM. Each datapoint represents a single mouse. PLX, PLX5622; VEH, vehicle; SH, sham; STR, stroke.

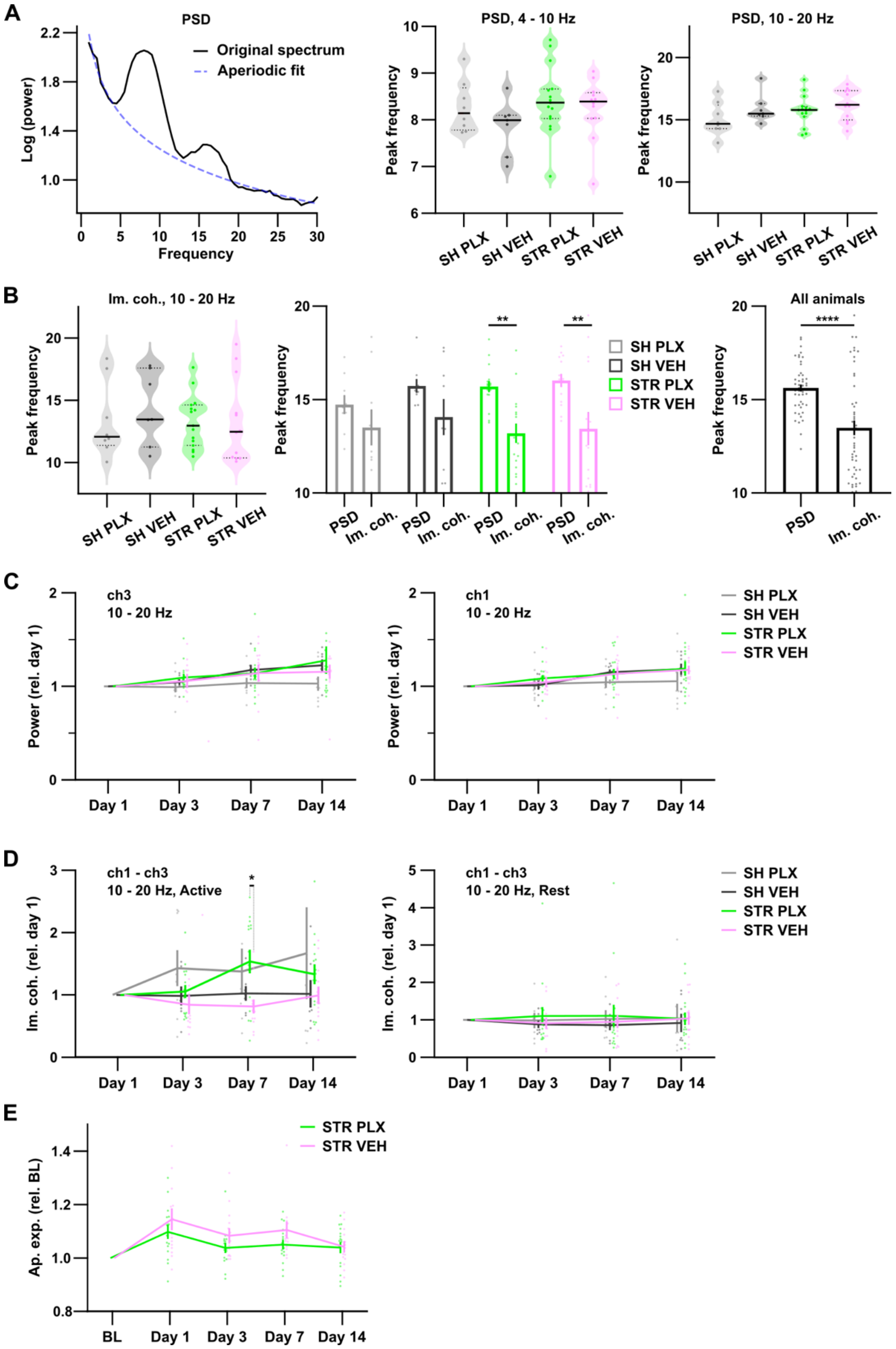
A
PSD
Original spectrum
Aperiodic fit
Log (power)
Frequency
PSD, 4 - 10 Hz
PSD, 10 - 20 Hz
Peak frequency
SH PLX
SH VEH
STR PLX
STR VEH
B
Im. coh., 10 - 20 Hz
PSD
Im. coh.
All animals
C
ch3
ch1
10 - 20 Hz
Power (rel. day 1)
Day 1
Day 3
Day 7
Day 14
D
ch1 - ch3
10 - 20 Hz, Active
10 - 20 Hz, Rest
Im. coh. (rel. day 1)
E
Ap. exp. (rel. BL)
BL

Supplementary Figure 5. Various supporting analyses. **A**, Left, example power spectrum (PSD) illustrating the original spectrum compared to the aperiodic fit. Middle and right, violin plots depicting theta and beta peak frequencies in all four groups during active behavior in ch3 (M1) at baseline. **B**, Left, violin plot depicting peak frequency of ch1-ch3 (M1) imaginary coherence. Middle, bar graph comparing M1 beta peak frequencies in PSD vs. imaginary coherence in all four groups. Right, direct comparison of M1 beta peak frequency for all animals. **C**, The fold-change of 10-20 Hz power in ch1 (left) and ch3 (right) in relation to day 1 are shown for all four groups. **D**, The fold-change of ch1-ch3 10-20 Hz imaginary coherence during active (left) and resting (right) periods of the experiment are depicted for all animals in relation to day 1. **E**, Direct comparison of fold-change of ch3 (M1) aperiodic exponents in both stroke groups are shown in relation to baseline. Sample sizes were: **A**, **B left**, **C, D left**: SH PLX: n = 8; SH VEH: n = 7; STR PLX: n = 15; STR VEH: n = 12. **B middle**: SH PLX: n = 9; SH VEH: n = 9; STR PLX: n = 16; STR VEH: n = 14. **D right**: SH PLX: n = 7; SH VEH: n = 7; STR PLX: n = 15; STR VEH: n = 12. **E**: STR PLX: n = 15; STR VEH: n = 12. **B right**: n = 48 total. Each datapoint represents a single mouse. Data were analyzed using two-way ANOVA with Šidák's multiple comparisons test in **A, B left, B middle, E**; two-way ANOVA with Tukey's multiple comparisons test in **C, D**; and Welch's t-test in **B right**. * $p<0.05$, ** $p<0.01$, *** $p<0.001$. Data are shown ± SEM. SH, sham; STR, stroke; PLX, PLX5622; VEH, vehicle.

Supplementary Tables

Supplementary Table 1. Stereotactic coordinates of implanted electrodes and corresponding neuroanatomical area

Electrodes	Coordinates (in relation to bregma, mm)		Neuroanatomy
	Anterior-posterior	Medio-lateral	
ch1 & ch2	+2	± 1.5	MOp (MOs)
ch3 & ch4	+1.5	± 2.5	MOp
ch5 & ch6	+1	± 1.5	MOp
ch7 & ch8	-0.5	± 2.5	SSp
ch9 & ch10	-1	± 1.5	SSp
ch11 & ch12	-1.5	± 3.5	SSp
ch13 & ch14	-2	± 2.5	SSp
ch15 & ch16	-3	± 1.5	VISam

MOp = primary motor area (M1), MOs = secondary motor area (M2), SSp = primary sensory area (S1), VISam = anteromedial visual area.

Supplementary Table 5. Various correlation pairs

	Repeated measures correlation		
**Variable 1**	**Variable 2**	**r**	**p**
Im. coh., ch1-ch3, 10-20 Hz	Ap. exp., ch3	0.286	0.119
Im. coh., ch1-ch3, 10-20 Hz	Rel. power, 10-20 Hz, ch1	-0.131	0.483
Im. coh., ch1-ch3, 10-20 Hz	Rel. power, 10-20 Hz, ch3	0.007	0.969
Im. coh., ch4-ch14, 10-20 Hz	Rel. power, 10-20 Hz, ch4	0.63	0.743
Im. coh., ch4-ch14, 10-20 Hz	Rel. power, 10-20 Hz, ch12	0.001	0.998
Im. coh., ch3-ch9, 4-10 Hz	Rel. power, 4-10 Hz, ch3	-0.434	0.019
Im. coh., ch3-ch9, 4-10 Hz	Rel. power, 4-10 Hz, ch9	-0.345	0.067
Im. coh., ch6-ch12, 30-60 Hz	Rel. power, 30-60 Hz, ch6	0.335	0.070
Im. coh., ch6-ch12, 30-60 Hz	Rel. power, 30-60 Hz, ch12	0.242	0.198
Ap. exp., ch3	SPR performance	-0.108	0.569

Repeated-measures correlation, r- and p-values. Imaginary coherence (relative to day 1; im. coh.), relative power (relative to overall 1-60 Hz power and relative to day 1; rel. power), aperiodic exponents (relative to baseline; ap. exp.) and SPR performance (relative to baseline) in the PLX-treated stroke group. n = 14-16 depending on channel combination.
